# Incidence and Heritability of Gastric Carcinoma in the Belgian Shepherd Dog Population in The Netherlands

**DOI:** 10.3390/vetsci12010018

**Published:** 2025-01-04

**Authors:** Sanne Hugen, Citlalli Limpens, Joris H. Robben, Hille Fieten, Paul J. J. Mandigers

**Affiliations:** 1 Department of Clinical Sciences of Companion animals, Faculty of Veterinary Medicine, Utrecht University, 3584 CL Utrecht, The Netherlands; c.limpens@uu.nl (C.L.); j.h.robben@uu.nl (J.H.R.); h.fieten@uu.nl (H.F.); p.j.j.mandigers@uu.nl (P.J.J.M.); 2 Evidensia Referral Hospital Arnhem, 6825 MB Arnhem, The Netherlands

**Keywords:** stomach cancer, population genetics, tervueren, Belgian sheepdog, groenendael

## Abstract

Background: Gastric carcinoma in the Tervueren and Groenendael varieties of the Belgian Shepherd dog is commonly recognized and carries a grave prognosis. Information on incidence and heritability is necessary to design an effective selection strategy against this disease. Methods: A database of cases and controls was built including cases with Tier 1 (confirmed via endoscopy or post-mortem) and Tier 2 evidence of gastric carcinoma. From the database, a birth cohort of dogs born in the Netherlands in the period 2000–2010 was used, together with pedigree information to calculate heritability using a mixed models approach. Univariate linear regression was used to test the influence of the gastric carcinoma status of the parents on gastric carcinoma occurrence in offspring. Results: For Tervueren the 10-year cumulative incidence was 4.7%, and for the Groenendael this was 2.1%. There was no sex predilection. The odds of gastric carcinoma were 5.0 (2.4–11.0) in offspring where at least one parent was known to be affected versus at least one parent was known to be unaffected. Heritability, based on analysis using pedigree information from 338 dogs with gastric carcinoma and 159 controls, was 0.53 (SE 0.15). Conclusions: Gastric carcinoma in the Belgian Shepherd Tervueren and Groenendael varieties has a high incidence in the Dutch population and a strong genetic component. Because it is a late onset disease, dogs have often already been used for breeding before they are diagnosed. An effective breeding strategy for reducing disease incidence therefore relies on the identification of genetic risk factors influencing its development.

## Simple Summary

Gastric carcinoma is a type of cancer with a strong breed predisposition in the Tervueren and Groenendael long-haired varieties of the Belgian Shepherd dog. We conducted a study of the incidence of gastric carcinoma in Tervueren and Groenendael dogs that were born in the Netherlands between 2000 and 2010. We found that on average during this period 3.8% of long-haired Belgian Shepherds develop gastric carcinoma during their lifetime. For the Tervueren variety the percentage of reported dogs that developed gastric carcinoma during their lifetime was 4.7% and for the Groenendael 2.1%. No sex predilection was demonstrated. The median age at death from gastric carcinoma in these dogs was 9.0 years. The heritability was calculated to be 0.53, indicating a strong hereditary component to development of the disease. The odds of developing gastric carcinoma in offspring are higher if at least one parent is affected compared to if parents are not known to be affected. Effective selection against this disease in this dog population is challenging, as dogs are often diagnosed with this disease after they have been used for breeding.

## 1. Introduction

Gastric carcinoma (GC) is rare in the general dog population, corresponding to less than 1% of all neoplastic changes identified [1]. It is diagnosed at a median age of 8.5 to 10 years through endoscopy confirmed via histological evaluation of tissue biopsies [2,3,4,5]. No specific hematological abnormalities or biomarkers have yet been identified to diagnose GC in dogs [6]. Surgery, with or without adjuvant chemotherapy, is the only potential curative treatment. However, prognosis even with surgery is very poor, with a median survival time of 33–178 days [7,8,9]. The diagnosis is often delayed by the non-specific, and initially mild clinical signs. Gastric carcinoma has even been diagnosed without overt clinical signs in Belgian Shepherds [10].

The etiology of GC in dogs is unknown. However, breed predispositions to GC have been reported, suggesting a genetic component to the etiology [1,2,3,11]. The Belgian Shepherd breed in Europe consists of four varieties; the Malinois, Lakenois, Groenendael, and Tervueren, and inter-variety crossing may be allowed. In the Tervueren and Groenendael varieties of the long-haired Belgian Shepherd dog a strong breed predisposition to GC not present in the Malinois or Lakenois has been reported [12,13,14,15].

Although the breed predisposition for GC in Belgian Shepherds indicates a genetic cause for the disease, currently no strong information about the heritability of the disorder is available. Globally, Belgian Shepherd breeders are seeking tools to decrease the frequency of GC in their breed. In order to predict the effectiveness of selection against GC in breeding strategies, knowledge on age distribution, heritability, and incidence of the disease in the population is necessary. Also, risk assessment for GC based on the disease status of one or both of the parents may help in decision-making for breeding strategies.

Therefore, the aim of the current study was to determine age, sex distribution, and incidence, to calculate the odds ratios for developing GC in offspring from affected parents, and to determine heritability of GC in the Belgian Shepherd’s Tervueren and Groenendael population.

## 2. Materials and Methods

### 2.1. Case and Control Definition and Recruitment

A database of GC cases was built by including all cases of GC, regardless of breed variety or country of residence, seen as a result of referral to one of the authors (PM) for gastroscopy and subsequent confirmation via histology. Additionally, all cases of GC in Dutch pure-bred Belgian Shepherds reported to the primary investigator (SH) by dog owners directly, cooperating pathology laboratories, or via the GC contact point of the Dutch Belgian Shepherd breed association (Nederlandse Vereniging voor Belgische Herdershonden, NVBH) between 2013 and 2024 were included. For these cases the medical records were requested and evaluated if available to assess the GC diagnosis.

A Tier 1 diagnosis of GC was defined as GC confirmed via endoscopy or post-mortem. A Tier 2 diagnosis was assigned to cases with incomplete or unavailable records and those based on ultrasonography or clinical presentation.

Belgian Shepherds without gastro-intestinal signs above the age of 10 years were actively recruited via the breed organization and the primary author and included in a database of possible control dogs. Health status was followed up through phone calls, e-mail, and questionnaires via the breed association and the primary investigator until death. Dogs that were followed until death and died without gastrointestinal signs above the age of 13 years were used as control dogs for this study.

The full pedigree database of Belgian Shepherds in the Netherlands was obtained via the Dutch Belgian Shepherd Club (NVBH) and adjusted to include only the long-haired varieties and Malinois with long-haired offspring.

### 2.2. Age and Sex Distribution of Gastric Carcinoma

Median and range of age of GC death were calculated for all cases with a reported date or age of death, per breed variety and per sex. Student’s t-test was used to test the difference in age of death by breed variety or sex and the chi-squared test for a difference in sex distribution.

### 2.3. Incidence

Belgian Shepherds with gastric carcinoma living in the Netherlands and born between 2000 and 2010 were selected from the GC database. The incidence was defined as the percentage of GC cases per birth year born in the Netherlands, and the cumulative incidence as the average incidence over these years. Incidence was calculated by dividing the total number of positive cases per birth year by the total number of Tervueren and Groenendael dogs born and registered in the Netherlands per year.

### 2.4. Calculation of Odds Ratios for Affected Parents

A univariable logistic regression model was used to calculate the odds ratio for an individual being affected depending on the disease status of the parents. For this analysis, the only dogs included from the case and control database were those for which at least one parent was also reported to be either case or control. The disease status of the other parent could be unknown. Odds ratio and 95% confidence interval were reported.

### 2.5. Heritability

All Dutch GC cases in Tervueren and Groenendael dogs that were bred with a Fédération Cynologique Internationale (FCI) pedigree were included in the heritability analysis. Dogs with a non-Dutch pedigree living outside of the Netherlands were only included in the heritability calculation if they were a first-line relative of a Dutch GC case. Dogs with an unknown phenotype were not used for the heritability calculations but were taken into account when calculating relatedness.

Narrow-sense heritability was calculated for the GC as a binary trait (affected and free). Using R core commands (R version 4.1.2), the dogs registered in the dataset with a phenotype and their parental data were filtered from the dataset. The additive relationship matrix of the dogs was calculated using the package optiSel [16]. Using the Sommer package a linear mixed models approach was used with random effects based on the additive relationship matrix to calculate the genetic and residual variances [17]. Narrow-sense heritability was then calculated using the following formula:

$$H^{2}=\frac{Genetic\;variance}{Genetic\;variance+Residual\;variance}$$

## 3. Results

### 3.1. Cases and Controls

The full database of GC cases contained 383 Belgian Shepherds. Of these dogs, 343 were Tervueren or Groenendael born in or living in the Netherlands, or were first line relative to a Dutch Tervueren or Groenendael. Only three Malinois and no Lakenois cases were present in the GC database.

**Figure 1:**
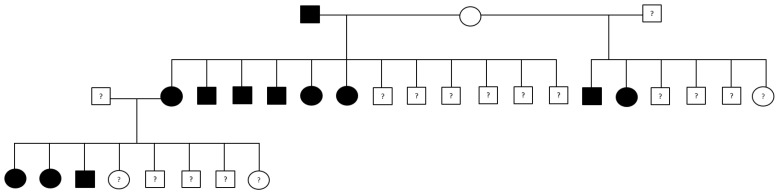
Visualization of a family tree including dogs affected by gastric carcinoma, controls, and animals with unknown phenotype, illustrating gastric carcinoma in multiple generations and multiple affected dogs per litter. Males are represented by squares, females by circles. Affected dogs are black, control dogs white. Unknown phenotype is indicated by a question mark.

Five of the Tervueren and Groenendael dogs had no reliable pedigree data and were excluded from analysis, leaving a study dataset of 338 GC cases, spanning multiple generations (Figure 1). A total of 216 cases (64%) had Tier 1 evidence, and 122 (36%) had Tier 2 evidence of GC.

A total of 320 potentially healthy control dogs were entered into the database of the GC research. Of these, 159 (112 Tervueren, 47 Groenendael) met the criteria of being Dutch and having no gastrointestinal signs before death at 13 years or older. The mean age at death of this group was 14.7 years (range 13.0–17.4).

### 3.2. Age and Sex Distribution of Gastric Carcinoma

**Figure 2:**
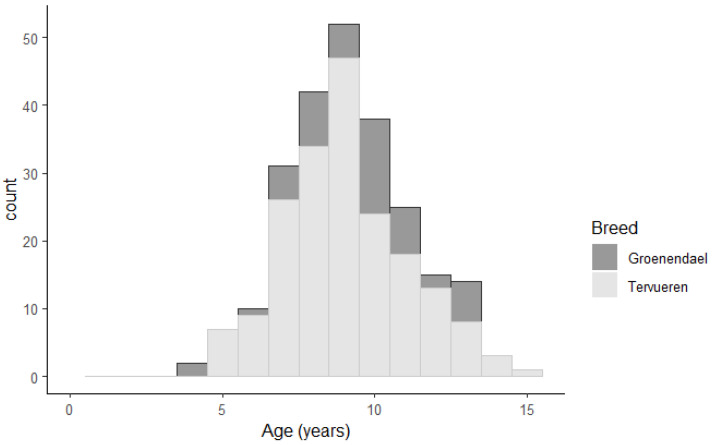
Age of death due to gastric carcinoma in a cohort of the long-haired Belgian Shepherd dog population in the Netherlands (born 2000–2010).

Age of death from GC was available from 240 cases (71% of all cases). The median age of death was 9.0 years (4.4–15.5). Age of death related to GC was not significantly different for males and females or Tier 1 and Tier 2 GC evidence, and not significantly higher for the Groenendael cases (9.7 years) compared to the Tervueren (9.0 years, *p* = 0.1, Figure 2). A total of 11 cases were older than 13 years at death due to GC (3% of Tier 1 cases, 7% of Tier 2 cases).

There was no sex predilection to GC in the study dataset with 169 male cases and 169 female cases.

### 3.3. Incidence

A total of 224 long-haired Belgian Shepherds in the research dataset were born between the years 2000 and 2010. In the same period, 5929 pups were born and registered, which means that the cumulative incidence of GC in the Belgian Shepherd long-haired varieties is 3.8%. For Tervueren the 10-year cumulative incidence is 4.7%, for Groenendael 2.1%. The incidence per year varies between 0% and 5.6% for the Groenendael and 2.4% and 8.4% for the Tervueren (Table 1).

### 3.4. Calculation of ORs for Affected Relatives

Of all dogs with GC in the study cohort, 30% had at least one litter in the Netherlands. For 137 dogs from the case or control group, the GC status of either dam or sire or both was known. The dam phenotype was known for all of these, the father phenotype for 67 dogs.

The odds of getting GC are significantly higher if at least one parent is affected versus no (known) parents are affected (OR 4.98, 95% CI 2.41–11.0) (Table 2).

### 3.5. Heritability

The entire available pedigree of Tervueren and Groenendael consisted of 19,494 dogs, bred between 1949 and 2024, spanning 23 generations. The heritability of GC in the narrow sense was calculated as 0.53 with a standard error of 0.15.

## 4. Discussion

Gastric carcinoma is very common in the Belgian Shepherd Tervueren and Groenendael varieties and carries a grave prognosis [4,7,18]. The median age of death from GC reported here is similar to previous reports, but with a larger range [18]. A possible explanation could be that owners of older dogs with suspected GC do not seek diagnosis confirmation and they are therefore not included in studies based on histopathology or endoscopy only. This is also reflected in the higher percentage of cases older than 13 years in the Tier 2 evidence group. In this study we used age of death instead of age at diagnosis as age at diagnosis was not available for all cases. However, given the short median survival time for GC in the Belgian Shepherd, age of death can be used to infer age of occurrence [18].

In contrast to smaller studies in the past suggesting a sex predisposition of males, this study confirms our previous results that there is no sex predisposition to GC in the Belgian Shepherd [1,4,18,19].

The increased prevalence for GC in the Belgian Shepherd has been described in several countries including the US, the Netherlands, Italy, Finland, and Norway [1,12,13,15,19]. The first GC incidence inventory in Dutch Tervueren was previously performed over the years 1991–2002. This study only incorporated 92 dogs with 50 known pedigrees. The reported incidence per year based on this study varied between 0.31 and 2.2% [19]. The higher incidence reported in this more comprehensive study (2.4–8.4%) could be explained by the more pro-active and better organized collection of GC cases by the investigators in close cooperation with the breed association. A true increase in incidence cannot be excluded however. An underestimation of the true incidence remains possible, as cases can still be missed.

The incidence of GC varied during the 10-year study period, but was overall high for both Tervueren and Groenendael varieties. One reason for the ongoing high incidence of GC in the Tervueren and Groenendael is because age of occurrence is much higher than the breeding age. This is reflected in the high percentage of GC cases that have produced offspring. Nine dogs affected with GC in this dataset even had two affected parents. The odds of being affected with GC are almost five times higher with at least one parent affected compared to at least one parent with a control dog status.

The high heritability of 0.53 is an additional strong indication for a genetic component to GC in this breed. Missing heritability may be attributable to environmental factors such as dietary influences. This will need to be further explored in future studies.

Not all family relations can be taken into account in the heritability calculation, as only animals with known phenotypes were used to construct the genetic additive matrix. We chose to only include controls if above the age of 13, and without any GI signs. Having strict inclusion criteria for controls contributes to the number of dogs with ‘unknown’ status. As these may have been missed cases, this could lead to an underestimation of heritability. On the other hand, having an underrepresentation of control dogs may bias to an overestimation of heritability.

Other limitations of this study are that not all GC cases had a Tier 1 diagnosis definitively confirming GC and not all patient files were available on the older cases. Abdominal ultrasound can be highly indicative of GC, especially loss of wall layering [20,21]. However, ultrasound is not very sensitive for GC, missing up to 50% of cases [22]. An additional limitation of this study for calculation of the heritability and OR for affected parents, was that not all parental status was known. Including more data would make it possible to calculate the additive effect of multiple parents affected. If grandparent status was available for more animals, it could perhaps be possible to select based on these.

A striking point and unique feature of the Belgian Shepherd breed is that only two of the four breed varieties are highly affected by GC. This is reflected in the fact that over a 15-year period only three Malinois and no Lakenois were identified with GC, even though the Malinois is the far more popular variety with annual registrations of nearly three times the number of pups per year compared to the Tervueren and five times for the Groenendael. This fact holds both promise for finding genetically protective factors for GC and implies the possibility of reducing disease prevalence using Tervueren born from Malinois or pure Malinois or Lakenois in breeding programs. In the absence of genetic screening possibilities, selection for reduction of GC in the Tervueren and Groenendael is at the moment nearly impossible. To decrease the incidence in the population, breeding from affected parents should be avoided. One possibility would be to only use semen from older males and freezing sperm to ensure genetic availability of dogs that are unaffected above 13 years. As selection based on first-line relatives affected does not appear possible at the moment, there is a strong need for the identification of causal mutations or associated variants that can be used in selection of breeding stock. Future research should therefore focus on the genetic factors underlying GC risk. Additionally, ongoing screening and inventory of all breeding dogs is necessary for the Tervueren and Groenendael to build more multigenerational data to strengthen future analysis and breeding advice. A veterinary practice disease-monitoring tool such as the PetScan system in the Netherlands may be a good addition to avoid underreporting due to lack of referral [23].

## Figures and Tables

**Table 1:** Gastric carcinoma incidence per birth year in a cohort of Dutch long-haired Belgian Shepherds (born 2000–2010).

Year	No. of Cases Tv */Gr *	Total Dogs Born Tv/Gr	Affected Tv (%)	Affected Gr (%)	Affected Tv + Gr (%)
2000	9/4	316/191	2.8	2.1	2.8
2001	13/9	365/266	3.8	3.4	3.6
2002	10/8	324/255	3.4	3.1	3.3
2003	13/7	315/289	4.1	2.4	3.5
2004	36/5	430/253	8.4	2.0	6.0
2005	24/10	321/180	8.1	5.6	7.2
2006	16/2	258/168	6.2	1.2	4.2
2007	17/3	304/218	5.3	1.4	3.6
2008	12/2	352/223	3.7	0.9	2.6
2009	12/3	244/207	5.3	1.4	3.5
2010	7/0	288/162	2.4	0.0	1.6
Cumulative	273/65	3517/2412	4.7	2.1	3.8

* Tv; Tervueren, Gr; Groenendael.

**Table 2:** Number of affected versus unaffected individuals for each combination of parent case/control status in a cohort of Dutch long-haired Belgian Shepherd dogs (born 2000–2010).

	Affected Dogs (*n* = 98)	Unaffected Dogs (*n* = 39)
One parent affected, other unknown/unaffected	46 (46.9.%)	7 (17.9%)
Both parents affected	9 (8.7%)	0 (0%)
One parent unaffected, other unknown	43 (41.3%)	32 (82%)

## Data Availability

The data presented in this study are available on request from the corresponding author due to privacy reasons.
